# Opsoclonus-myoclonus syndrome attributable to West Nile encephalitis: a case report

**DOI:** 10.1186/1752-1947-8-232

**Published:** 2014-06-26

**Authors:** Victoria Bîrluţiu, Rareş Mircea Bîrluţiu

**Affiliations:** 1 Faculty of Medicine Sibiu, “Lucian Blaga” University Sibiu, Alba-Iulia Str. No.79 23/8, Sibiu 550052, Romania

**Keywords:** Encephalitis, Opsoclonus-myoclonus syndrome, West Nile virus

## Abstract

**Introduction:**

Opsoclonus-myoclonus syndrome is a very rare neurological disorder associated with some viral infections and exceptionally with the West Nile virus.

**Case presentation:**

A 57-year-old Caucasian woman presented with fever, dizziness, balance difficulties, vomiting, dancing eye, altered speech, tremor, generalized myoclonus and failure to rise or stand. Our objective is to describe a patient with West Nile infection, which was identified both in her serum and cerebrospinal fluid and was associated with encephalitis and opsoclonus-myoclonus-ataxia syndrome.

**Conclusions:**

Opsoclonus-myoclonus-ataxia syndrome continued for 4 weeks after onset, when she died. There was no evidence for any other etiology responsible for her opsoclonus-myoclonus syndrome. Her opsoclonus-myoclonus syndrome appeared associated with West Nile encephalitis and had an unfavorable evolution despite treatment.

## Introduction

Opsoclonus-myoclonus syndrome (OMS) also known as the dancing eye syndrome is a very rare neurological disorder, affecting 1 in 10,000,000 people per year. In children, OMS was associated with neuroblastoma; in adults it may be associated with breast carcinoma or small-cell lung carcinoma, uterus or ovarian cancers, rare gall bladder, pancreas and renal cell carcinoma. It can also be consequent to toxic medication (amitriptyline, haloperidol, diazepam), metabolic disorders [1], hydroelectrolytic disorders, brain anoxia or autoimmune cause – Hashimoto's encephalopathy [2], autoantibodies against neurons and cerebellar Purkinje cells [3], celiac disease, and one of the paraneoplastic syndromes. Although it is not considered an infectious disease, it can be associated with some viral infections, which may trigger the debut of OMS [4], such as the Epstein–Barr virus, coxsackie B2, B3 virus, Saint Louis encephalitis virus, human immunodeficiency virus [1], hepatitis C infection [5], rubella, mumps [6], varicella-zoster virus [7], and West Nile virus [8]. OMS was reported as a poststreptococcal infection associated with antibodies against a 56 kDa protein [9], in adolescents following an infection with *Mycoplasma pneumoniae*[10], *Rickettsia* and as a manifestation of acute *Borrelia burgdorferi* infection [11] and sometimes with vaccination. The conventional therapies with corticosteroids, intravenous immunoglobulin, plasmapheresis, adrenocorticotropin hormone, antiepileptic drugs are associated with long-term neurological morbidity; the combination of cyclophosphamide and dexamethasone pulse therapy [12] or rituximab administered in the refractory cases are promising [13,14]. The best treatment in adults is not very clear. Steroids, baclofen, clonazepam, intravenous immunoglobulin, and immune-adsorption therapy are used currently [15].

We report a case of opsoclonus-myoclonus syndrome associated with West Nile encephalitis.

## Case presentation

Our patient was a 57-year-old Caucasian woman who had an acute onset of dizziness 2 days prior to presentation to hospital. She began to have fever, 38.8°C, vomiting, diarrhea, and sleepiness. Her past medical history was dominated by larynx neoplasm (surgically eradicated 4 years before) and high blood pressure. She was not on any medications. On admission she presented a respiratory rate of 26 breaths/minute, arterial oxygen saturation 93%, heart rate 72 beats/minute, blood pressure 120/80mmHg, opsoclonus with rapid, involuntary, multivectorial, conjugated fast eye movements persisting during sleep, no modification of her visual field, generalized myoclonus (brief, involuntary twitching of muscles), and she was unable to perform the finger to nose test; sleep disturbance, altered speech, mild neck stiffness, positive Kernig's sign, abnormal plantar responses and diminished osteotendinous reflexes. Work up included blood test and spinal fluid analysis, which are summarized in Tables 1 and 2 (only pathological results). Paraneoplastic antibodies anti-Hu, −Ri, −Yo were negative.

**Table 1:** Cerebrospinal fluid studies

**C**e**rebrospinal fluid**	**Results**
Nucleated cells	49/mm^3^ – 75% lymphocytes, 25% granulocyte
Glucose	115mg/dL
Protein	0.429g/L
Gram smear	No organism
Culture	No growth
West Nile virus immunoglobulin M	Positive

**Table 2:** Serum studies

**Serum**	**Results**
Glucose	236mg/dL
Leukocytes	12,000/mm^3^
	Granulocytes 79.7%, Ly 14.8%, Mxd 5.5%,
Hemoglobin	15g/dL
Hematocrit	42.4%
West Nile virus immunoglobulin M 2nd serum sample	Positive, increase in evolution
Lyme disease immunoglobulin M	Negative

A cranial native computed tomography scan examination was performed and showed infra and supratentorial focused ischemia lesions.

The conclusions drawn from brain magnetic resonance imaging were: cortical and cerebellum atrophy; lacunary infarcts and bilateral infra-tentorial and over-tentorial demyelinating lesions; and ethmoid sinusitis.

She was given intravenous dexamethasone, symptomatic treatment with clonazepam, cerebral depletion treatment (such as administration of intravenous solutions like mannitol), supportive therapy with intravenous fluid, and antibiotics to prevent other types of bacterial infection. She had persistent OMS. After 2 weeks she presented an acute retention of urine and 4 weeks after admission she died. A postmortem examination did not reveal any malignancy, systemic diseases or toxic etiology.

## Discussion

The West Nile virus is a mosquito-borne zoonotic arbovirus of the *Flaviviridae* family that is found in temperate and tropical regions. It can cause a serious neurological illness in less than 1% of infected people: encephalitis, meningoencephalitis, acute flaccid paralysis – poliomyelitis-like, Guillain–Barré syndrome and optic neuritis. In Romania, in 1996, the West Nile was responsible for a high number of neuroinvasive diseases. In recent years there has been a sporadic spread in this area, and a few cases of meningitis or encephalitis have been recorded annually. This is the first West Nile encephalitis with OMC diagnosed in our country. OMC may occur transiently in hyperglycemic coma but our patient presented hyperglycemia only on the first day after admission and the West Nile infection was shown both in her serum and cerebrospinal fluid (CSF) examination. Opsoclonus can be associated with severe evolution to an immunocompromised host. Her CSF examination was associated with a low CSF pleocytosis of 49 leukocytes/mm^3^ with 25% neutrophilic pleocytosis, more characteristic of West Nile neuroinvasive disease than most other common forms of viral encephalitis. CSF glucose was higher because the blood glucose levels were higher.

## Conclusions

In our patient the OMS appeared associated with West Nile encephalitis and had an unfavorable evolution despite treatment with corticosteroids. It is essential to combine available treatments but the evolution is less satisfactory.

## Consent

Written informed consent was obtained from the patient’s next of kin for publication of this case report and any accompanying images. The study was accepted by the Ethics Committee of the hospital and they encouraged us to publish the article. A copy of the written consent is available for review by the Editor-in-Chief of this journal.

## Abbreviations

CSF: Cerebrospinal fluid; OMS: Opsoclonus-myoclonus syndrome.

## Competing interests

The authors received no funding for the manuscript preparation or for publishing the article. The authors declare that they have no competing interests.

## Authors’ contributions

VB made substantial contribution to conception and design of the manuscript. VB also performed the analysis and interpretation of data. RB was involved in drafting the manuscript and acquisition of data. Both authors read and approved the final manuscript.
